# Synergistic Tribo-Activity of Nanohybrids of Zirconia/Cerium-Doped Zirconia Nanoparticles with Nano Lamellar Reduced Graphene Oxide and Molybdenum Disulfide

**DOI:** 10.3390/nano10040707

**Published:** 2020-04-08

**Authors:** Dinesh Kumar Verma, Nivedita Shukla, Bharat Kumar, Alok Kumar Singh, Kavita Shahu, Mithilesh Yadav, Kyong Yop Rhee, Rashmi Bala Rastogi

**Affiliations:** 1 Department of Chemistry, Indian Institute of Technology, Banaras Hindu University, Varanasi 221005, India; dineshkv.rs.chy15@iitbhu.ac.in (D.K.V.); niveditashukla.rs.chy17@itbhu.ac.in (N.S.); bharatkr.rs.chy16@itbhu.ac.in (B.K.); alokkrsingh.rs.chy17@itbhu.ac.in (A.K.S.); kavita.rs.chy17@itbhu.ac.in (K.S.); 2 Department of Chemistry, Prof. Rajendra Singh (Rajju Bhaiya) Institute of Physical Sciences for Study & Research, V.B.S. Purvanchal University, Jaunpur 222003, India; dryadavin@gmail.com; 3 Department of Mechanical Engineering, College of Engineering, Kyung Hee University, Yongin 446-701, Korea

**Keywords:** doped nanoparticles, nanohybrids, X-ray photoelectron spectroscopy, friction, wear, tribo-activity

## Abstract

Zirconia and 10%, 20%, and 30% cerium-doped zirconia nanoparticles (ZCO), ZCO-1, ZCO-2, and ZCO-3, respectively, were prepared using auto-combustion method. Binary nanohybrids, ZrO_2_@rGO and ZCO-2@rGO (rGO = reduced graphene oxide), and ternary nanohybrids, ZrO_2_@rGO@MoS_2_ and ZCO-2@rGO@MoS_2,_ have been prepared with an anticipation of a fruitful synergic effect of rGO, MoS_2_, and cerium-doped zirconia on the tribo-activity. Tribo-activity of these additives in paraffin oil (PO) has been assessed by a four-ball lubricant tester at the optimized concentration, 0.125% w/v. The tribo-performance follows the order: ZCO-2@rGO@MoS_2_ > ZrO_2_@rGO@MoS_2_ > ZCO-2@rGO > ZrO_2_@rGO > MoS_2_ > ZrO_2_ > rGO > PO. The nanoparticles acting as spacers control restacking of the nanosheets provided structural augmentation while nanosheets, in turn, prevent agglomeration of the nanoparticles. Doped nanoparticles upgraded the activity by forming defects. Thus, the results acknowledge the synergic effect of cerium-doped zirconia and lamellar nanosheets of rGO and MoS_2_. There is noncovalent interaction among all the individuals. Analysis of the morphological features of wear-track carried out by scanning electron microscopy (SEM) and atomic force microscopy (AFM) in PO and its formulations with various additives is consistent with the above sequence. The energy dispersive X-ray (EDX) spectrum of ZCO-2@rGO@MoS_2_ indicates the existence of zirconium, cerium, molybdenum, and sulfur on the wear-track, confirming, thereby, the active role played by these elements during tribofilm formation. The X-ray photoelectron spectroscopy (XPS) studies of worn surface reveal that the tribofilm is made up of rGO, zirconia, ceria, and MoS_2_ along with Fe_2_O_3_, MoO_3_, and SO_4_^2−^ as the outcome of the tribo-chemical reaction.

## 1. Introduction

Interfacial friction between the surfaces in relative motion is of great concern as it causes energy loss due to liberated frictional heat. The phenomenon is accompanied by loss of mass, too, in the form of surface wear. A lubricant interposed between the moving surfaces minimizes the interfacial friction and wear. Energy conservation and environmental protection are vital issues regarding the growth of the industry in the world today. Selection of an energy-efficient lubricant that can ameliorate the fuel efficiency and impede emissions may prove pivotal in addressing these issues [1,2,3]. Nanomaterials, due to their high chemical stability and small size, are being preferred as lubricant additives over the conventional organics [4,5,6,7]. The small size of nanomaterials triggers fast tribological action at the surface while their chemical stability minimizes emissions [8,9,10]. Wide varieties of nanoparticles, like metal oxides, metal sulphides, metal halides, and carbon-based materials, have been reported in the literature as lubricant additives [11,12,13,14,15,16]. Doping by some different metal in the host lattice of a metal salt has yielded enhanced tribo-performance due to the formation of defects [17,18,19]. Doping of zinc oxide by magnesium or aluminum is known to improve its tribological properties [17]. Zinc-doped calcium copper titanate has been reported by our laboratory to give good activity [18]. Doping of zirconia by magnesium, calcium, aluminum, yttrium, etc. has enhanced its tribological properties [18]. Mixed-metal oxide nanopowders containing ceria-zirconia and alumina have been reported for catalytic activities [20]. Philip et al. [21] have studied surface morphology and stability analysis of ceria-zirconia hybrid nanoparticles. Based on X-ray diffraction (XRD) results, they have proposed an fcc-fluorite structure and suggested their application as a lubricant additive.

Inorganic two-dimensional layered nanomaterials, like molybdenum disulfide and graphene, have accentuated research on account of their distinctive properties, mainly high specific surface area [3,22,23,24]. In the layered structure, weak van der Waals-type interactions exist between adjacent layers [8,25]. This arrangement confers exceptional mechanical, thermal, electrical, and optical properties to such materials [26,27,28]. Therefore, their applications in various fields, like catalysis, electronics, photonics, energy storage, and sensors, have been fully acknowledged [29]. Their vital role in lubrication is attributed to the very low friction coefficient because of facile shearing of nanosheets under sliding motion [27,30,31,32,33]. Graphene sheets consist of carbon atoms in the sp^2^-hybridized state, forming a honeycomb-type of lattice. The large surface area, along with high thermal conductivity and excellent mechanical stiffness, make graphene a significant material in tribological applications [3].

MoS_2_ possesses a hexagonal close-packed structure in which each molybdenum atom is covalently bonded to six sulfur atoms in a trigonal prismatic fashion. The presence of weak van der Waals forces between molecular S–Mo–S tri-layers facilitates interlayer slippage. MoS_2_ and graphene nanosheets or their nanocomposites have been used very often as oil additives for enhanced lubrication [34,35,36]. Xu et al. [35] observed synergic tribological behavior of MoS_2_ and graphene dispersed in esterified bio-oil. The tribological activity of hydrothermally synthesized composite, MoS_2_-graphene oxide, has been reported by Song and coworkers [36].

Wu and his associates have investigated tribological properties of the chemically capped zinc borate/MoS_2_ nanocomposite in oil and grease [37,38]. Tribological behavior of Fe_3_O_4_/MoS_2_ nanocomposite in water/oil was studied by Zheng et al. [39]. Recently, Fe_3_O_4_/MoS_2_ nanocomposite was reported to show enhanced lubricating properties in base oils together with significant photocatalytic degradation [40]. The nanocomposite of WS_2_ with graphene was reported to yield better tribological results than its constituents [41]. A nanohybrid of boron, nitrogen, co-doped graphene with titania was found to reduce friction, wear tremendously, and enhance load-carrying properties [42]. Recently, reinforcement of reduced graphene oxide (rGO) by magnesium-doped zinc oxide showed wonderfully exalted tribological behavior over pure zinc oxide [43]. It was thought to add up the effects of doped nanoparticles, rGO and MoS_2,_ together in the form of a tailor-made nanocomposite to obtain captivating tribo-performance.

Zirconia in the tetragonal phase is a hard, wear-resistant material with high chemical stability and load-bearing capacity [21]. For the furtherance of its wear resistance and load-bearing capacity, an attempt may be made to stabilize the same phase by doping with ceria. Since the drastic reduction in friction and wear has been claimed in case of graphene supplemented with ceria [44] or zirconia [45], it appealed to us to prepare cerium-doped zirconia nanoparticles using the auto-combustion method [43]. Thus, nanoparticles were prepared, and the existence of the tetragonal phase was identified by their X-ray diffraction patterns [19,21]. These nanoparticles have been used for preventing agglomeration of rGO. Molybdenum disulfide nanosheets have been prepared by hydrothermal method using ammonium heptamolybdate [40]. The nanohybrids were prepared to have the accretive effect of zirconia/cerium-doped zirconia, reduced graphene oxide, and molybdenum disulfide on the enhancement of lubrication.

As-prepared nanoparticles, nanosheets, and nanohybrids were characterized by scanning electron microscopy/high-resolution scanning electron microscopy (SEM/HR-SEM), energy dispersive X-ray analysis (EDX), powder X-ray diffraction (XRD), Fourier transform infrared microscopy (FTIR), Raman, and electronic (UV-visible) spectroscopy.

## 2. Materials and Methods

### 2.1. Chemical and Reagents

Extra-pure ZrO(NO_3_)_2_·H_2_O, Ce(NO_3_)_3_·6H_2_O, (NH_4_)_6_Mo_7_O_24_·4H_2_O, and SC(NH_2_)_2_ were obtained from Sigma-Aldrich (Mumbai, India). Graphite flakes and potassium permanganate were received from NGS Naturgraphit GmbH (Leinburg, Germany) and Fluka (Shanghai, China), respectively. The other analytical reagent (AR)-grade chemicals used in the present investigations, like anhydrous citric acid, ammonia solution, hydrazine hydrate, hydrogen peroxide, sulphuric, and phosphoric acid, were obtained from Merck (Delhi, India).

### 2.2. Synthesis of Nano-Additives

#### 2.2.1. Preparation of Zirconia and Cerium-Doped Zirconia Nanoparticles, 10% (ZCO-1), 20% (ZCO-2), and 30% (ZCO-3)

Synthesis of ZrO_2_ and 10%, 20%, 30% cerium-doped ZrO_2_ nanoparticles ZCO-1, ZCO-2, and ZCO-3, respectively, were carried out by auto-combustion method [43]. For the preparation of ZrO_2_, at first, a homogeneous aqueous solution of ZrO(NO_3_)_2_·H_2_O and (7.54 g) and citric acid (3.76 g) was made in 30 mL of distilled water. It was heated at nearly 230 °C with continuous stirring to yield the gel. Further heating of the gel resulted in the formation of ash. The blackish ash was calcined at 500 °C in a muffle furnace for 2 h to yield ZrO_2_ nanoparticles. Similarly, 10%, 20%, 30% cerium-doped zirconia ZCO-1, ZCO-2, and ZCO-3 nanoparticles were synthesized by adding the required amount of ZrO(NO_3_)_2_·H_2_O, Ce(NO_3_)_3_·6H_2_O, and citric acid.

#### 2.2.2. Preparation of Graphene Oxide (GO) and Reduced Graphene Oxide (rGO)

The graphene oxide (GO) and reduced graphene oxide (rGO) were prepared by modified Hummer’s method with the help of microwave as reported earlier [43].

#### 2.2.3. Preparation of MoS_2_ Nanosheets

For the preparation of MoS_2_ nanosheets, hydrothermal method was used [38]. At first, (NH_4_)_6_Mo_7_O_24_·4H_2_O (2.40 g) and SC(NH_2_)_2_ (5.20 g) were dissolved in 80 mL of distilled water by ultrasonication for 30 min to get a uniform solution. The prepared solution was poured into a 200-mL Teflon autoclave, and the temperature was adjusted to 180 °C for 12 h. The formed product was cooled down to room temperature (RT). It was washed by a water-ethanol mixture and dried in a vacuum oven at 60 °C.

#### 2.2.4. Preparation of Binary Composites of ZrO_2_/ZCO-2 with Reduced Graphene Oxide

The ZrO_2_ nanoparticles (700 mg) and rGO nanosheets (467 mg) were dispersed in 100 mL ethanol separately by using ultrasonicator for 1 h at RT. The dispersions were intermingled by continuous stirring and sonicated at 70 °C. The mixture was kept for 5 min in the microwave oven at 700 W to yield the black-color product [40].

The binary nanocomposite ZCO-2@rGO was synthesized by the same methodology.

#### 2.2.5. Preparation of Ternary Composites of ZrO_2_/ZCO-2, rGO and MoS_2_

Molybdenum disulfide 700 mg was dispersed in 100 mL of ethanol. The prepared dispersion was added to the mixture of ZrO_2_/ZCO-2 and rGO. The ternary composites were prepared from this reaction mixture following the same procedure as that for binary composites.

**Figure 1:**
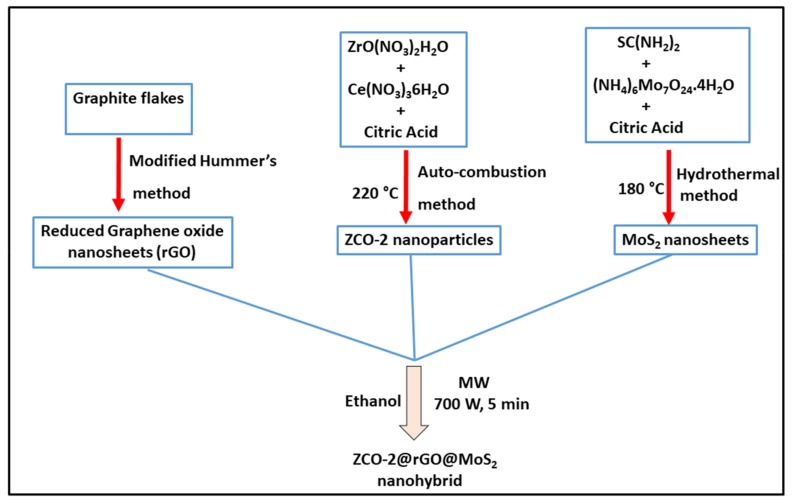
Schematic representation for the procedure of synthesis of ternary nanohybrid ZCO-2@rGO@MoS_2._

A schematic representation for the procedure of synthesis of ZCO-2@rGO@MoS_2_ nanohybrid is shown in Figure 1.

### 2.3. Techniques Employed for Characterization of the Nano Additives

For studying morphological features of the nanoparticles, nanosheets, and their nanohybrids, scanning electron microscopy/high resolution scanning electron microscopy (HR-SEM) using FEI-Nova Nano SEM 450 (FEI, Hillsboro, OR, USA) and transmission electron microscopy (TEM)/high-resolution transmission electron microscopy (HR-TEM) using FEI-Tecnai-G2 electron microscope (FEI, Hillsboro, OR, USA) were carried out. Confocal Micro-Raman mapping system (UniRAM, employing excitation wavelength, 785 nm laser) was utilized for taking Raman spectra. X-ray diffraction (XRD) (Rigaku Miniflex 600, XRD-System, Hong Kong, China) using Cu-Kα_1_ radiation (λ = 1.54 Å) and X-ray photoelectron spectroscopy (XPS) (PHI 5000 Versa Probe II, FEI, Inc., Hillsboro, OR, USA) were employed for investigation. FTIR spectra were recorded on a Thermo Scientific Nicolet iS5 FTIR spectrometer (Waltham, MA, USA).

### 2.4. Tribological Tests

Paraffin oil (PO) used in the present study possessed similar characteristic properties as reported in our previous investigations [43]. Its blends with the studied additives were prepared in various concentrations, 0.0625%, 0.1250%, 0.1875% and 0.2500% (w/v) by sonicating at RT for 1 h. As per test results, the concentration 0.125% w/v was found to be the optimized concentration. The tribological tests, therefore, were conducted at 0.125% w/v concentration of all the additives in base lube, MoS_2,_ rGO, ZrO_2_, ZCO-1, ZCO-2, ZCO-3, ZrO_2_@rGO, ZCO-2@rGO, ZrO_2_@rGO@MoS_2_, and ZCO-2@rGO@MoS_2_. Steel balls (American Iron and Steel Institute (AISI) 52100 alloy, hardness: 59–61 Hardness Rockwell C (HRC), and diameter: 12.7 mm) were used during present work. Pre- and post-cleaning of the balls was performed using n-hexane through ultra-sonication followed by drying.

Four-Ball Lubricant Tester (Ducom Instruments Pvt. Ltd., Bangalore, India) was used to study tribological behavior of PO and its admixtures with different nano additives following the conditions of ASTM-D4172 and ASTM-D5183 tests. The diameter of wear scars appearing on three lower stationary balls was measured, and their mean was shown as mean wear scar diameter (MWD). For determination of wear rate, the test was conducted at 392 N load for 1.5 h duration and values of MWD were noted after every 15 min test run. From MWD data, the corresponding values for mean wear volume (MWV) were obtained. The details about the tribological parameters are described in Supplementary Materials Figure S1.

Structural features of the wear track supplemented with blank oil with and without additives were studied by SEM and atomic force microscopy (AFM). The ZEISS, EVO-Scanning Electron Microscope MA 18 (Oberkochen Germany) was employed to take SEM images of the wear track of the steel balls. For surface roughness measurement, AFM (Nova Px 3.2.5 NT-MDT, Moscow, Russia) was used.

## 3. Results and Discussion

### 3.1. Structural and Morphological Features of Nano Additive

The characterization of synthesized nanoparticles zirconia/cerium-doped zirconia ZCO-2, their corresponding nanohybrids with reduced graphene oxide (ZrO2/ZCO-2@rGO), and ternary nanohybrids including molybdenum disulfide, (ZrO2/ZCO-2@rGO@MoS_2_) were successfully brought about by XRD, FTIR, UV/visible, Raman, TEM/HR-TEM, SEM/HR-SEM, and XPS techniques.

**Figure 2:**
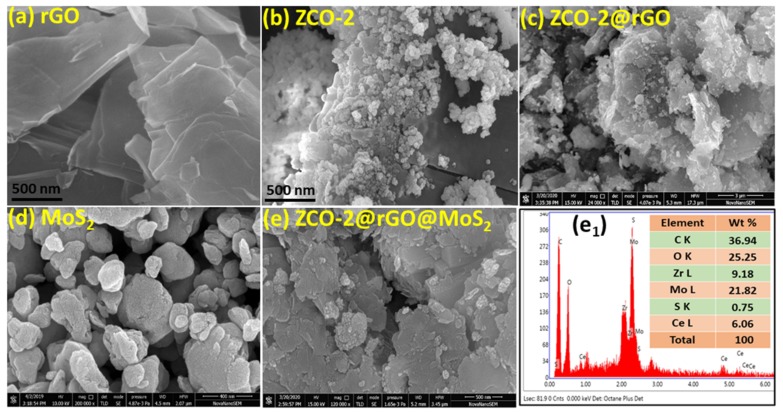
High resolution scanning electron microscopy (HR-SEM) images of (**a**) rGO, (**b**) ZCO-2, (**c**) ZCO-2@rGO, (**d**) MoS_2_ and (**e**) ZCO-rGO@MoS_2_ with its EDX spectrum (**e**_1_).

The HR-SEM images of rGO nanosheets, ZCO-2 nanoparticles, the nanohybrid ZCO-2@rGO, MoS_2_ nanosheets, and the ternary nanohybrid, ZCO-2@ rGO@MoS_2,_ are presented in Figure 2a–e, respectively. Figure 2a exhibits a lamellar structure for rGO [42,43]. The crumpled and transparent sheets of rGO with folded edges are visible. Figure 2b shows ZCO-2 nanoparticles of almost spherical shape. However, a slight agglomeration of the nanoparticles can be seen. Figure 2c presents the graphene sheet adorned with ZCO-2 nanoparticles. Its EDX Elemental mapping is provided in Supplementary Materials Figure S1a to show the distribution of ZCO-2 nanoparticles (NPs) on rGO nanosheets. The EDX spectrum of ZCO-2@rGO (not given here for the sake of brevity) shows elemental composition. Presence of clearly visible peaks assignable to zirconium, cerium, and oxygen, in addition to carbon, is indicative of the formation of the proposed nanohybrid. Figure 2d displays nanosheets of MoS_2_ while Figure 2e illustrates MoS_2_ nanosheets attached on rGO layers and ZCO-2 nanoparticles are decorated over them. The EDX spectrum of the ternary nanohybrid provides perspicuous signals for the elements molybdenum and sulfur, in addition to zirconium, cerium, oxygen, and carbon. The depicted wt. % data in Figure 2e_1_ endorses the formation of ternary nanohybrids. EDX elemental mapping is given in Figure S1b to differentiate rGO, MoS_2,_ and ZCO-2 in the nanocomposite.

**Figure 3:**
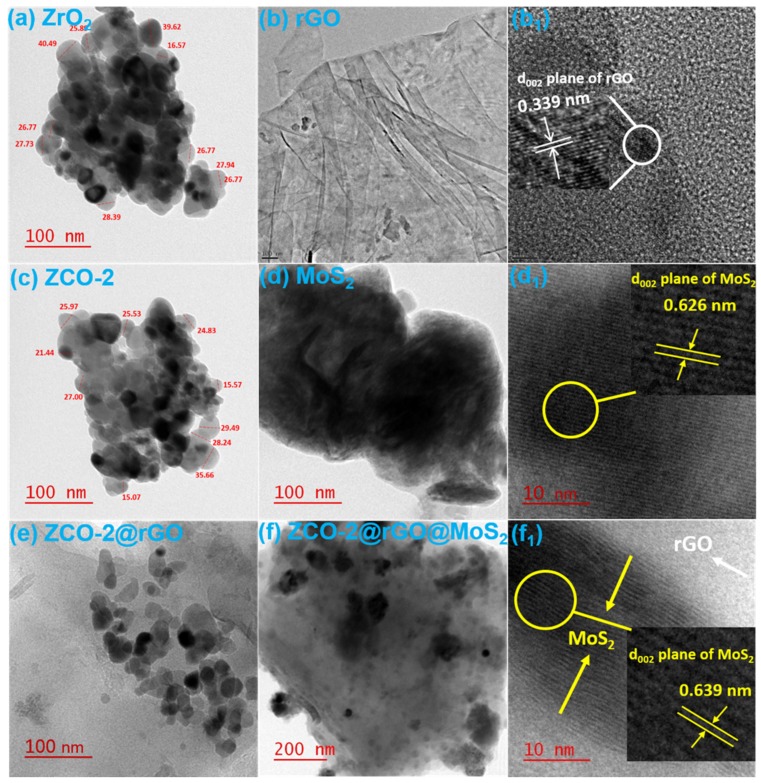
Transmission electron microscopy (TEM) images of (**a**) ZrO_2_, (**b**) rGO, (**c**) ZCO-2, (**d**) MoS_2,_ (**e**) ZCO-2@rGO, and (**f**) ZCO-2@rGO@ MoS_2_; and HR-TEM images of (**b**_1_) rGO, (**d**_1_) MoS_2,_ and (**f**_1_) ZCO-2@rGO@ MoS_2_.

For a deeper understanding of morphological characteristics, TEM images of the studied nanomaterials were also recorded and are displayed in Figure 3, ZrO_2_ (a), rGO (b), ZCO-2 (c), MoS_2_ (d), ZCO-2@rGO (e), and ZCO-2@rGO@MoS_2_ (f), respectively. HR-TEM images of rGO, MoS_2_, and ZCO-2@rGO@MoS_2_ are also presented in Figure 3b_1_,d_1_,f_1_, respectively. The tetragonal phase of ZrO_2_ and ZCO-2 nanoparticles is apparent in the TEM images (Figure 3a,c) [19,20]. The average size of a nanoparticle seems to be in the range of 25–30 nm. The lamellar structure of rGO is noticeable from Figure 3b. The nanosheets appear to be quite transparent with little folding at the edges. The interlayer distance of (002) plane of rGO was obtained as 0.339 nm [30,42,43]. The HR-TEM image of MoS_2_ nanosheets showed a layered structure with weak van der Waals-type of forces existing in between the layers. The interlayer distance of the (002) plane, 0.62 nm was in concurrence with XRD. The findings regarding the microstructure of MoS_2_ nanosheets were the same as reported by Rawat et al. [28,46]. However_,_ the TEM and HR-TEM images of the ternary nanohybrids, ZCO-2@rGO@MoS_2,_ reveal that MoS_2_ nanosheets were scattered on the surface of rGO. The nanoparticles ZCO-2 gracing the rGO and MoS_2_ surfaces are conspicuously visible in Figure 3f. Presence of sharp, polycrystalline rings, along with strong diffraction spots in the selected area diffraction pattern (SEAD) of the ternary nanohybrids (shown in the inset of Figure 3f), is indicative of high crystallinity. It is a noticeable feature of HR-TEM Figure 3f_1_ that the interlayer distance of MoS_2_ nanosheets [28] remarkably enhanced to 0.639 nm [3] in the ternary nanohybrid. Thus, it may be inferred that the nanoparticles acted as spacers [30,44,45] between rGO and MoS_2_ layers and alleviated their restacking [8,14,28,30,44,45].

**Figure 4:**
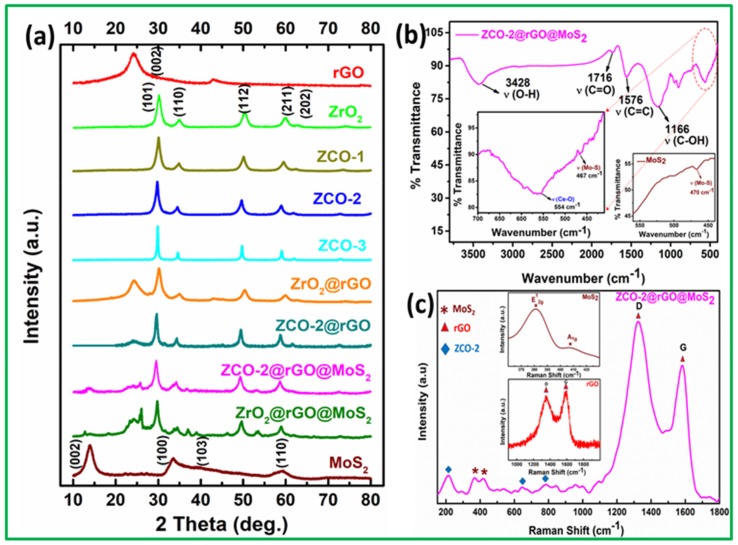
(**a**) X-ray diffraction (XRD) patterns of as-prepared nano additives, (**b**) Fourier transform infrared spectroscopy (FTIR), inset showing υCe-O and υMo-S and, (**c**) Raman spectra of ternary composite ZCO-2@rGO@MoS_2_ (inset showing spectra of MoS_2_/rGO).

Figure 4a displays X-ray diffraction (XRD) patterns of rGO, ZrO_2_, ZCO-1, ZCO-2, ZCO-3, ZrO_2_@rGO, ZCO-2@rGO, MoS_2_, ZrO_2_@rGO@MoS_2,_ and ZCO-2@rGO@MoS_2_. Reduction of GO to rGO is apparent as the typical peak of GO appearing around 11° due to (001) reflection disappears in XRD of rGO and the nanohybrids. The diffraction patterns of rGO and the nanohybrids exhibit a broad peak at 24.5° assignable to (002) reflection of rGO [43].

The diffraction pattern of ZrO_2_ is indicative of tetragonal crystallites [19]. The tetragonal structure continued to exist in cerium-doped zirconia ZCO-1, ZCO-2 and ZCO-3, as apparent from their diffraction patterns. Absence of any additional peak due to cerium confirms that a single tetragonal phase was maintained. As compared to pure zirconia, the intensity of the peaks reasonably decreased in the case of cerium-doped zirconia. However, the diffraction patterns of the nanohybrids ZrO_2_@rGO and ZCO-2@rGO showed the same peaks but with comparatively much-reduced intensity.

The XRD pattern of MoS_2_ can be indexed to the hexagonal structure as per JCPDS no. 37-1492 and JCPDS 77-1716 showing characteristic peaks at 14.3°, 33.6°, 39.4°^,^ and 59.4°, corresponding to (002), (100), (103), and (110) planes, respectively [28,39]. The presence of strong and sharp diffraction peak for (002) plane conformed to a well-stacked lamellar structure [28]. The d-spacing of the (002) plane was calculated as 0.626 nm, which matched exactly with the TEM analysis [28,46]. The phase structure of MoS_2_ was carried as such in the ternary nanohybrids with a difference that the intensity of the peak due to (002) plane was reduced sufficiently. The reduction in intensity indicates that crystallite size and number of layers along c axis were reduced in the nanohybrids as compared to MoS_2_ nanosheets. The interspacing of (002) plane exalted in ternary composites to 0.639 nm [3,30], indicating components in the hybrid well interacted with each other.

In the FTIR spectra (Figure 4b) of the nanohybrids, ZCO-2@rGO@MoS_2_, the absorption band due to Ce-O [21] and Mo-S [46] stretching frequencies were observed around 554 and 467 cm^−1^ besides typical bands of rGO [45] in the region 1100–1720 cm^−1^. The presence of these bands validates the formation of the ternary composite.

Raman spectra of rGO and its nanohybrids, employing 785 nm laser excitations, are shown in Figure 4c. There are two peaks of very high intensity at 1360 and 1582 cm^−1^ in the spectrum of rGO, which are attributed to D and G bands, respectively [43]. The D band is assignable to breathing mode of A_1g_ symmetry and provides information about sp^3^/sp^2^ hybridized defects. The G band is attributed to doubly degenerate phonon mode of E_2g_ symmetry and relates to vibration in the ordered sp^2^- hybridized carbon atoms. The intensity ratio of D and G bands *I_D_/I_G_* is an important parameter for interpretation of disorders or defects in the structure. Its value, 0.92 in rGO, increased to 1.34 in ZCO-2@rGO@MoS_2_, which may be associated with increased defects in the composite. For MoS_2_, E^1^_2g_ and A_1g_ bands were observed at 380 and 407 cm^−1^, respectively [28]. The E^1^_2g_ mode corresponded to in-layer displacement of molybdenum and sulphur atoms while A_1g_ mode related to out-of-layer symmetric displacement of sulphur atoms along the c axis. A difference of 27 cm^−1^ between these two modes indicates a multi-layered structure. The blue shift of A_1g_ and decrease of interpeak separation were significant for decreasing the number of MoS_2_ layers. These bands are marked in the Raman spectrum of the ternary nanohybrid. Several bands were also marked between 200 to 640 nm due to the tetragonal phase of zirconia [39].

### 3.2. Assessment of Tribological Behaviour of Nano Additives in Paraffin Oil

#### 3.2.1. Dispersion Stability

For determination of dispersion stability of different blends, absorbance values were recorded in the range 200–800 nm at six-hour intervals starting from zero up to 48 h using UV/visible spectroscopic technique. The samples for the test were prepared by 10-times dilution of the original dispersion containing the additives (0.125% w/v) in paraffin oil. Figure S2a illustrates the variation of relative absorbance vs. settling time for all nano additives. In every case, the relative absorbance dropped with time, but the dropping was beyond doubt least for the ternary nanohybrid ZCO-2@rGO@MoS_2_ followed by ZrO_2_@rGO@MoS_2_, then ZCO-2@rGO, ZCO-2, MoS_2_, and, at last, rGO.

Though the most active additive exhibited maximum stability, others also had adequate stability as relative absorbance dropped down only up to approximately 0.5. The inset of the figure shows the absorbance of ZCO-2@rGO@MoS_2_ within 48 h at 6 h intervals. It can be vividly seen that the composite absorbed at 320 nm and the absorbance decreased with time from 0.8 to approximately 0.6 within 48 h. Figure S2b exhibits the photographs of base oil and its dispersions with the ternary composite at zero time and after 48 h.

#### 3.2.2. Optimization of Concentration of Additives

**Figure 5:**
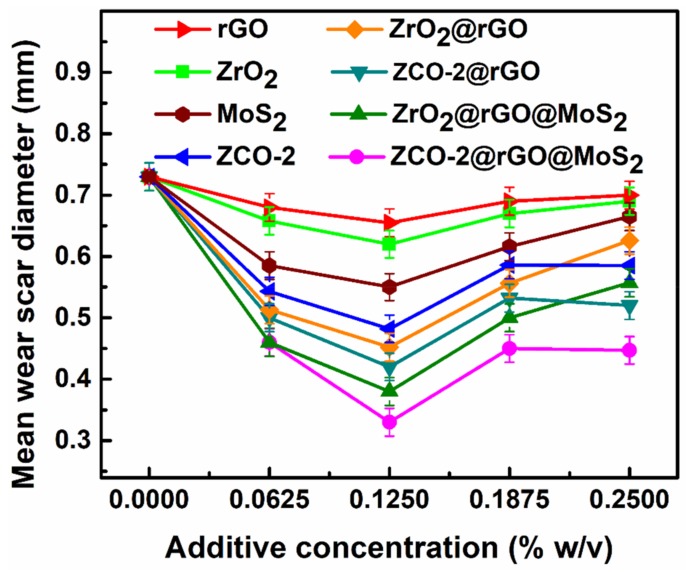
Variation of mean wear scar diameter for the paraffin oil as a function of additive concentration at 392 N applied load for 60 min duration.

The tests were performed to see the effect of concentration on tribological properties. The different concentrations of additives, starting from blank, 0.0625%, 0.1250%, 0.1875%, and 0.2500% w/v were tested at a load of 392 N for 60 min test duration in base lube, and corresponding results are displayed in Figure 5.

It can be easily stated that the tested formulations were found to be tribologically active at all the tested concentrations. At the first test concentration, 0.0625% w/v, MWD value for rGO fell off as compared to blank paraffin oil.

For different blends, the values of MWD were further lowered in the order ZrO_2_, ZCO-2, MoS_2,_ followed by binary nanohybrids ZrO_2_@rGO, ZCO-2@rGO, and, at last, ternary nanohybrids ZrO_2_@rGO@MoS_2,_ ZCO-2@rGO@MoS_2._ This sequence of diminution of MWD values in the presence of the investigated additives directly relates to the enhancement of their tribo-activity. For the next concentration, 0.1250% w/v, there was a further decline of MWD values for each of the additives and the same order concerning each other prevailed. When concentration was increased to 0.1875% w/v, the MWD values were tangibly increased in each case, although their mutual ordering remained the same. Consequent upon the remarkable increase of MWD values at 0.1875% w/v for all the tested additives, an inference was drawn that 0.1250% w/v should be taken as the optimized concentration for all the formulations. For the last concentration, 0.2500% w/v, MWD values were more or less stabilized or showed a slight increase in some cases.

#### 3.2.3. Friction and Wear-Reducing Properties

**Figure 6:**
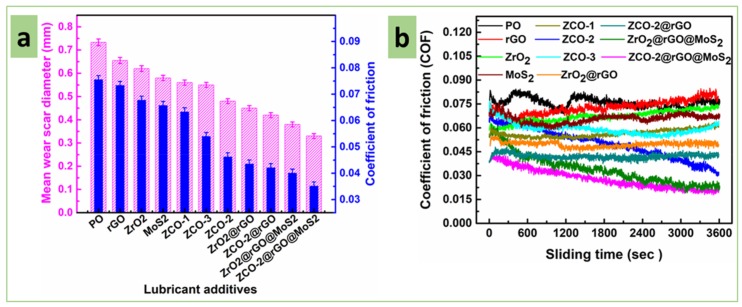
Variation in tribological parameters in the absence and presence of different nano additives (0.125% w/v) in paraffin oil: Load, 392 N; sliding speed, 1200 rpm; temperature, 75 °C; sliding duration, 60 min; concentration of additives, 0.125% w/v. (**a**) Mean wear scar diameter and the average coefficient of friction, (**b**) coefficient of friction as a function of sliding time.

For assessment of antiwear properties, ASTM-D4172 tests of base oil and its admixtures with different additives were conducted using the optimized concentration (0.1250% w/v) at 392 N load for 60 min test duration. The test results are summarized in Figure 6a. The figure shows the variation of the two most important parameters, mean wear scar diameter (MWD) and the average coefficient of friction (COF), together in the form of a bar diagram. It is clearly perceived from the figure that the base oil showed MWD as 0.733 mm, but in the presence of individual additives, percentage reduction increased gently, like rGO (11%), ZrO_2_ (15%), MoS_2_ (21%), ZCO-1 (24%), ZCO-3 (25%), and ZCO-2 (35%). The binary composites ZrO_2_@rGO and ZCO-2@rGO caused further increase in % reduction to 39 and 43, respectively. Indubitably, the humongous reduction was observed when ternary nanohybrids were used, ZrO_2_@rGO@MoS_2_ (48%) and ZCO-2@rGO@MoS_2_ (55%). Thus, illustrious antiwear behavior was observed in the case of the ternary composite of rGO and MoS_2_ with 20% cerium-doped zirconia (ZCO-2).

Likewise, the observed average COF value 0.0756 for plain paraffin oil underwent an enormous reduction in the presence of different additives following the same order as that of MWD, rGO (3%), ZrO_2_ (10%), MoS_2_ (13%), ZCO-1 (16%), ZCO-3 (29%) and ZCO-2 (40%), ZrO_2_@rGO (42%), ZCO-2@rGO (44%), ZrO_2_@rGO@MoS_2_ (47%), and ZCO-2@rGO@MoS_2_ (53%). The huge diminution in MWD and COF values in the presence of the above additives was directly related to their antiwear and antifriction properties, respectively. This kind of behavior may be interpreted in terms of the crucial role played by different additives in the formation of the tribochemical film (in situ), which prevented contact of the proximal surfaces and was indispensable for abatement of their friction and wear. Here nanoparticles as nano bearings and layered structure of rGO and MoS_2_ altogether facilitated the sliding motion, which in turn, ameliorated the efficiency of composites. Doping of zirconia by cerium increased the efficiency, which may be ascribed to created defects.

The Figure 6b portrays variation of COF of mating surface vs. time at 392 N load in plain base lube or its admixtures. It is a very important parameter from the viewpoint of life expectancy of machines. In general, COF values are initially high in each case. Later on, with time as tribofilm is formed, the values are reduced. It is apparent from the figure that the COF is inevitably highest for the PO alone. Its blend with rGO as additive comes next showing a fair amount of reduction in the COF values at the beginning of the test. However, after a certain period, little increase in COF values is noted probably due to slight agglomeration, which makes the surface less protected.

Further reduction in COF is identified when blends with zirconia nanoparticles followed by MoS_2_ nanosheets are implied. These nanomaterials are succeeded by doped nanoparticles in terms of reducing COF values. Out of 10%, 20%, and 30% cerium-doped zirconia nanoparticles (ZCO-1, ZCO-2, ZCO-3) as additives, the best results were obtained for ZCO-2 where the minimum values of COF are observed.

Further lowering of COF was visible when formulations consisting of binary composites were incorporated. A comparison of binary composite blends showed that COF was pretty low and consistent in the case of ZCO-2@rGO than ZrO_2_@rGO. Amazing results were noticed when ternary nanocomposites were appended to the base oil. Undoubtedly, ZCO-2@rGO@MoS_2_ outperformed ZrO_2_@rGO@MoS_2_ as discussed above for MWD values_._ Accordingly, out of all the tested additives, the lowest and stabilized COF was perceived in the case of ZCO-2@rGO@MoS_2_.

For determination of wear rate, the test was conducted at 392 N load for 1.5 h duration and values of MWD were noted after every 15 min test run. As mean wear volume (MWV) was a more justified parameter than MWD for calculation of wear rate, the observed MWD values for different sliding periods were converted to MWV using a suitable formula [43].

**Figure 7:**
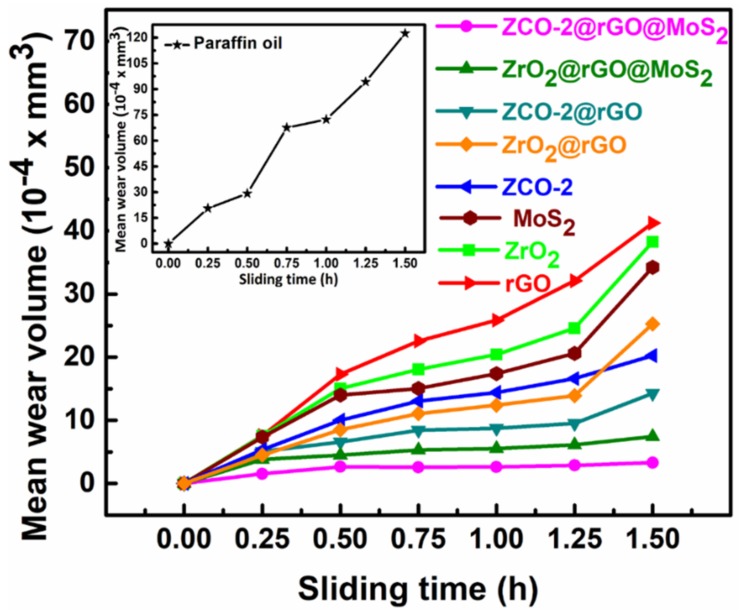
Variation of mean wear volume with sliding time for paraffin oil without (given in inset) and with 0.125% w/v of different nano additives for 1.5 h test duration.

Figure 7 portrays variation of MWV values as a function of sliding time from 0 to 1.5 h. Wear rate was obtained by fitting a linear regression model [43]. The running-in period was taken between 0–0.75 h while the steady-state period was considered during 0.75 h onwards.

Running-in and steady-state wear rates were obtained concerning the above mentioned periods and are depicted in Supplementary Materials Figures S3 and S4, respectively. The acquired data are presented in Table 1.

In the presence of plain base lube, the values of running-in and steady-state wear rates were quite high. However, these values underwent a severe reduction in the presence of blends of base oil with various additives.

The extent of reduction in wear rates for different additives was in tune with their antiwear properties, as discussed above. It is interesting to note that for ZCO-2 alone as an additive, the steady-state wear rate was significantly reduced. From the observed results of wear rates, it may be pleaded authoritatively that both the ternary composites, in general, and ZCO-2@rGO@MoS_2_, in particular, proved their potentiality for tribological applications.

#### 3.2.4. Load-Bearing Capacity

**Figure 8:**
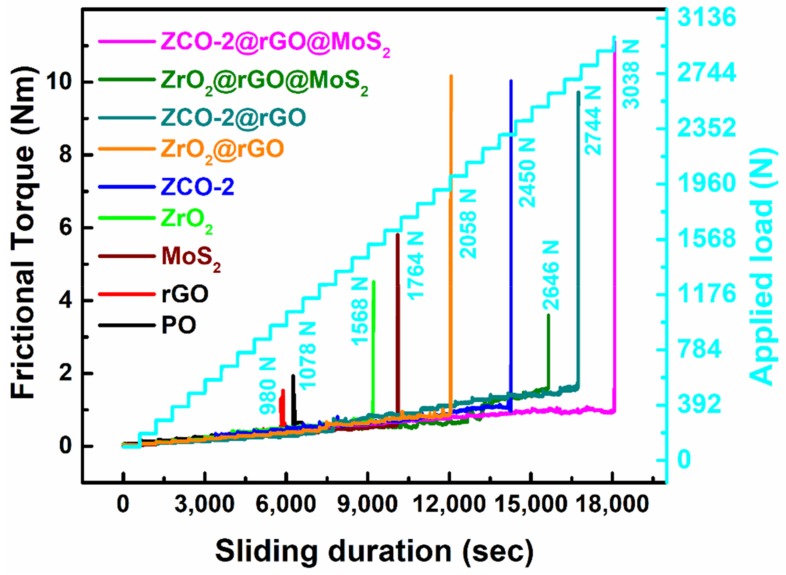
Variation of frictional torque as a function of stepwise loading and time for PO in absence and presence of different nano additives: Sliding speed, 600 rpm; temperature, 75 °C; concentration of additives, 0.125% w/v.

For determination of the load-bearing capacity of different admixtures, step loading test (ASTM D-5183) was conducted using the optimized concentration of the additives under the standard conditions of 392 N load, 600 rpm, 75 °C temperature, and 60 min duration, after the termination of the running-in period. The test for the steady-state coefficient of friction was carried out by subsequent increment of 98 N load after every 10 min. The observed data are presented in Figure 8. In the presence of plain paraffin oil alone, the value of frictional torque was quite high, and seizure of the tribo-pairs occurred at 1078 N. At the seizure load rupture of tribofilm occurred, which perturbed the steady-state. The lubricant lost its capability to bear the load. 

It is a noticeable feature of Figure 8 that plain base oil showed seizure load at 1078 N while its admixture with rGO sustained the load only up to 980 N. This unruly behavior of rGO was attributed to its weaker adhering tendencies. However, blends of base oil with other additives increase the seizure load ZrO_2_ (1568 N), MoS_2_ (1764 N), ZrO_2_@rGO (2058 N), ZCO-2 (2450 N), ZrO_2_@rGO@MoS_2_ (2646 N), ZCO-2@rGO (2744 N), and finally ZCO-2@rGO@MoS_2_ (3038 N). Thus, the presence of MoS_2_ and ZrO_2_ or ZCO-2 strengthened rGO, and maximum load-bearing capacity was observed for the ternary composite ZCO-2@rGO@MoS_2_.

#### 3.2.5. Morphological Features of the Worn Surface

**Figure 9:**
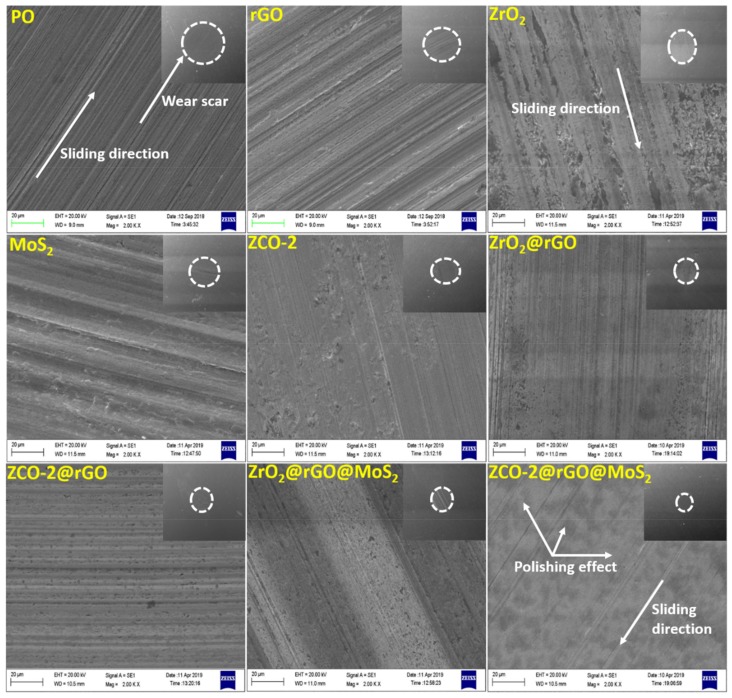
SEM micrographs (inset: Full view of wear scar at 100×, wear scar surface at 2000× magnification) of the worn steel surface lubricated with paraffin oil with and without different nano additives (0.125% w/v) for 60 min test duration at 392 N applied load.

The surface techniques, SEM and AFM, were employed for studying morphological characteristics of the wear track in the presence of paraffin oil alone or containing the investigated additives at the optimized concentration (0.125% w/v) under standard conditions of ASTM-D4172 test. The SEM images for base lube with or without considered nano additives are displayed in Figure 9. In the presence of base lube, the worn surface appears to be corrugated in the SEM images because of dreadful scratches. However, the worn surfaces are smoothened in the presence of the admixtures. The order of smoothening of the worn surfaces corroborated the antiwear properties of the additives. The values of MWD for base lube with or without additives is displayed in the inset of each micrograph. The value of MWD 0.733 mm for oil alone diminished significantly for its blends with different additives, rGO (0.655mm), ZrO_2_ (0.620mm), MoS_2_ (0.550mm), ZCO-2 (0.480mm), ZrO_2_@rGO (0.450mm), ZCO-2@rGO(0.420mm), ZrO_2_@rGO@MoS_2_ (0.380mm), and ZCO-2@rGO@MoS_2_ (0.330mm)). Descending order of MWD from plain oil through various additives to finally the ternary composite of ZCO-2 was harmonious with the gradual improvement of the surface. The individuals of the nanohybrids genuinely outperformed their part in achieving smoothness of the contact surfaces. The surface was mended by the nanoparticles as they were small enough to fill the minute pits or gaps [30,47]. The self-lubricating behavior of nanosheets further advanced as nanoparticles increased their dispersibility by preventing agglomeration [3,14,30].

**Figure 10:**
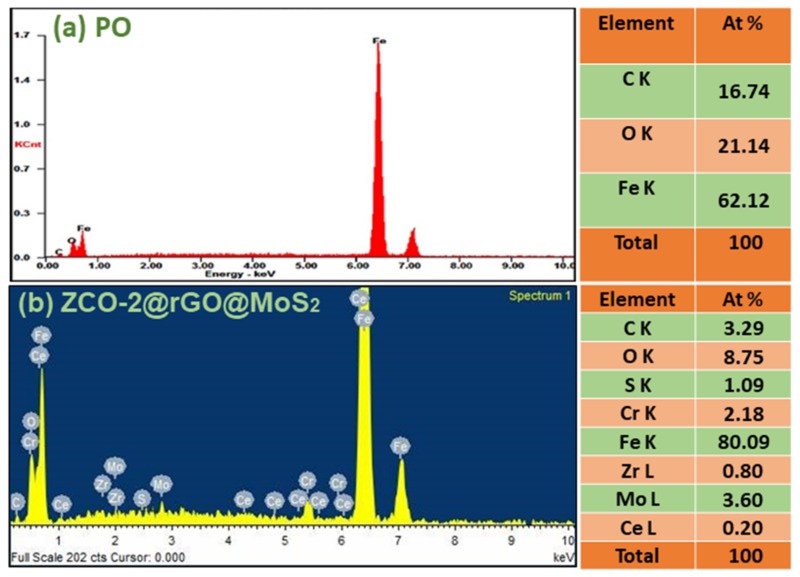
Energy dispersive X-ray (EDX) spectra of worn surface lubricated with (**a**) blank paraffin oil (PO) and (**b**) PO blended with 0.125% w/v ZCO-2@rGO@MoS_2_ nanohybrid at 392 N applied load.

The elemental mapping and EDX spectra of the tribofilm formed in the presence of a blend of ternary nanohybrid (ZCO-2@rGO@MoS_2_) with base oil at 392 N applied load shown in Figure S5 and Figure 10, respectively, provide information about its elemental composition. Besides the peaks in base oil (Figure 10a), some additional peaks due to zirconium, cerium, molybdenum, and sulfur are also observed in Figure 10b related to ternary nanohybrid, indicating the constituents of tribofilm.

**Figure 11:**
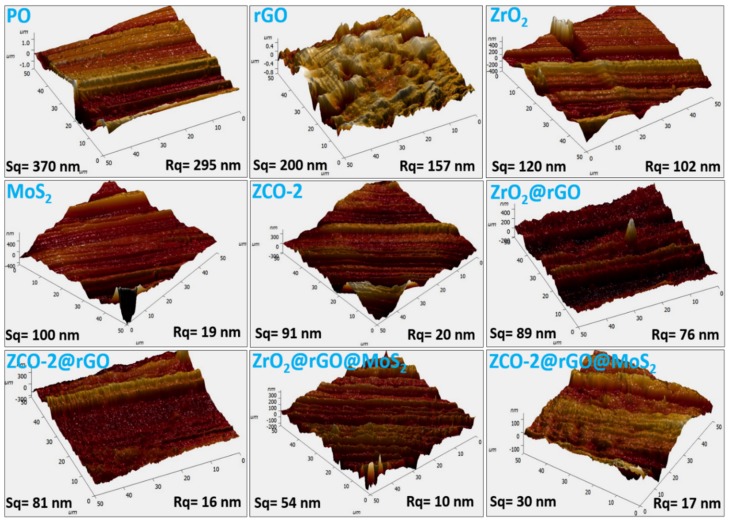
The 3-D atomic force microscopy (AFM) images of the worn steel surface lubricated with blank paraffin oil (PO) and blends of PO with 0.125% w/v nano additives at 392 N applied load.

Morphological characteristics of the surface of the balls after the antiwear test (392 N load, 1 h, 1200 rpm, 75 °C) performed in the presence of plain base oil and its blends with the synthesized additives were studied by AFM. The 3-D AFM images of the wear scar surface are presented in Figure 11, incorporating surface roughness values (line roughness (Rq) and area roughness (Sq)) as well.

It can be noticed that there is a substantial decrease in the values of Rq and Sq from plain paraffin oil to the blends of different investigated nano additives. For example, the Sq value of plain paraffin oil being 370 nm underwent 46% reduction in the presence of rGO nanosheets while in the case of nanoparticles reduction exceeded to 68% for ZrO_2_ and 75% for ZCO-2. The binary composites ZrO_2_@rGO and ZCO-2@rGO brought about further % reduction in Sq values to 76 and 78, respectively. Finally, surprising results for reduction in Sq values were obtained for the ternary composites ZrO_2_@rGO@MoS_2_ (85%) and ZCO-2@rGO@MoS_2_ (92%). The % reduction in surface roughness justifies the role of binary and ternary nanocomposites towards enhancing the lubricity of the base oil. The AFM images, thus, validate exactly the order obtained from tribological data and the related SEM images.

**Figure 12:**
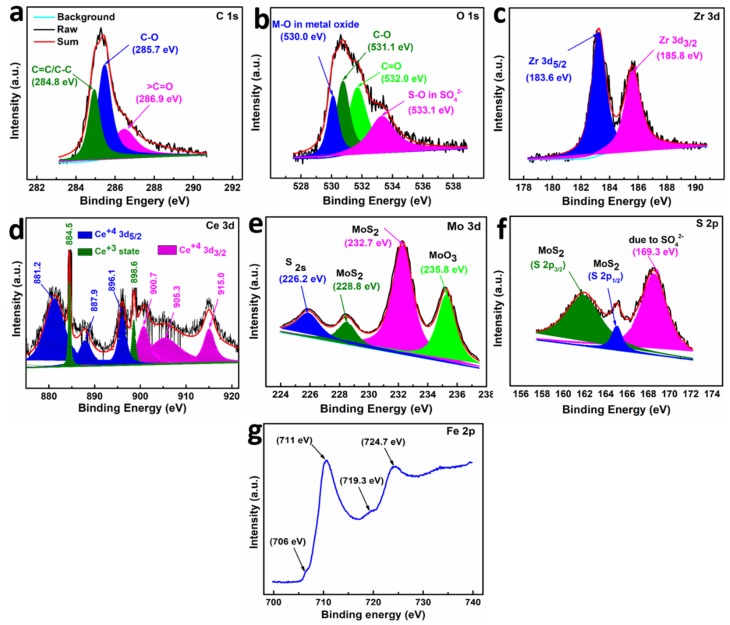
X-ray photoelectron spectroscopy (XPS) spectra of the worn surface lubricated with ZCO-2@rGO@MoS_2_ nanocomposite: (**a**) C 1s, (**b**) O 1s, (**c**) Zr 3d, (**d**) Ce 3d, (**e**) Mo 3d, (**f**) S 2p, and (**g**) Fe 2p spectra.

XPS studies were conducted to identify the chemical states of different elements in the tribofilm lubricated with ternary composite ZCO-2@rGO@MoS_2_. Use was made of XPS peak fit software to deconvolute the core level spectra. Figure 12a exhibits core level spectrum of C 1s deconvoluted into three peaks for C=C, C–O, and C=O bonds with corresponding binding energies 284.8, 285.7, and 286.9 eV, respectively [8,43,45]. The spectrum of O 1s displayed in Figure 12b shows three peaks. The peaks at 530.0, 531.1, 532.0, and 533.1 eV are indexed for M–O bonds of metal oxides [Zr(IV)–O, Ce(IV)/Ce(III)–O and Fe(II/III)–O], C–O, C=O, and –S(VI)–O bond of SO_4_^2−^, respectively [20,45,48,49]. The appearance of two peaks at 183.6 and 185.8 eV in the spectrum of Zr 3d (Figure 12c) assignable to Zr 3d_5/2_ and Zr 3d_3/2_, respectively, confirmed Zr in +4 state [45].

The presence of two sets of peaks in Ce 3d XPS spectrum (Figure 12d) signifies the presence of 3d_3/2_ and 3d_1/2_ states of Ce^4+^. The peaks with maxima at 900.7, 905.3, and 915 eV correspond to Ce^4+^ 3d_3/2_, whereas those at 881.2, 887.9, and 896.1 eV are ascribed to Ce^4+^ 3d_5/2_. Besides these peaks, two extra peaks with the extremely small area are observed at 884.5 and 898.6 eV, which may be attributed to traces of Ce^3+^ [20,48].

The intense peaks at 228.2 and 231.3 eV in Mo 3d spectrum (Figure 12e) are accorded with Mo 3d_5/2_ and Mo 3d_3/2,_ respectively. The additional peaks at 226.2 and 235.8 eV are ascribed to S 2s of MoS_2_ and Mo–O of MoO_3_, respectively [14,28,35]. Formation of MoO_3_ is attributed to oxygen substitution at the defects in MoS_2_ nanosheets [14,46]. The S 2p spectrum (Figure 12f) depicts two peaks of MoS_2_ at 162.3 and 163.9 eV for S 2p_3/2_ and S 2p_1/2_, respectively. Besides these peaks, an additional peak is also identified at 169.3 eV, depicting formation of sulphate [35,49]. The binding energies of Mo 3d and S 2p agree very well with the reported values for 2D MoS_2_ indicating chemical states as Mo^4+^ and S^2−^ [28]. The Fe 2p spectrum (Figure 12g) reveals Fe 2p_3/2_ and Fe 2p_1/2_ peaks of Fe_2_O_3_ at 711 and 724.7 eV, respectively. Xu et al. [49] showed that the peak at 724.7 eV also corresponds to iron sulfate. Besides, extremely weak peaks at 706, 719.3 eV denote that Fe is almost absent in the native state [49]. Thus, from XPS studies of the worn surface lubricated with the ternary composite ZCO-2@rGO@MoS_2_, it may be inferred that all the contents of the tribofilm including the organic residue of rGO which is adsorbed on the surface, metal oxides, MoS_2_, and products of tribo-reaction like iron sulphate, Fe_2_O_3,_ and MoO_3_ synergistically cooperated, yielding illustrious tribological performance of the ternary composite [50].

### 3.3. Tribo-Chemistry and Mechanism of Lubrication

Based on the above analytical data and discussion thereof, it may be deduced that compatibility of the investigated additives as antiwear agents is attributed to their adherence on the surface of the tribo-pairs which eventually leads to the formation of tribofilm under standard test conditions. The strong, persistent tribofilm formed in situ carries the load. The nature of tribofilm, in fact, plays a decisive role in the overall efficiency of the additives. The inconceivable efficiency of binary/ternary nanohybrids may be explained by invoking synergistic interaction between the individual components. The MoS_2_ and rGO mutually assisted each other during lubrication by maintaining their layered structures instead of getting smashed into small particles [3,35]. The presence of rGO helped in preventing the oxidation of MoS_2_ into MoO_3_ or sulfate [23,35].

Indubitably, the layered structure of rGO and MoS_2_ facilitated sliding motion, whereas nanoparticles anchored between nanosheets acted as spacers [30,44,45], and palliated their restacking [8,14,28,30,44,45]. Besides this, nanoparticles strengthened the nanosheets, prevented agglomeration, and increased their dispersibility [3,14,30,45]. They also contributed towards improving poor adherence of rGO [42].

Conversely, agglomeration of nanoparticles was also attenuated simultaneously by nanosheets. There is a possibility that these nanoparticles might have acted as nano bearings between nanosheets as well as proximal surfaces to reduce friction [5,14,15,30,51,52]. Thus, sliding motion was further facilitated, and lubrication was upgraded. Improvement of lubrication by nanoparticles was also achieved through their tribo-sinterization on the mating surfaces [5,30,51,52,53]. Consequently, small pits formed on the surface were restored by nanoparticles by mending mechanism [5,30,51,52,53]. The polishing effect of nanoparticles on the abraded surface was also important in enhancing lubrication [51,52]. A combination of the above mechanisms provided the excellent tribological performance of the nanocomposites [30].

Presence of ZrO_2_ provided hardness and toughness to the composite structure and assisted in enhancing its load-bearing capacity. Use of cerium-doped zirconia nanoparticles yielded better results as compared to zirconia nanoparticles themselves. The EDX and XPS studies of the wear scar surface lubricated with the best additive, ZCO-2@rGO@MoS_2_ nanohybrid, provided elemental composition and the chemical form of the constituents. The tribofilm composed of adsorbed rGO, MoS_2,_ zirconia, ceria, and the products of tribochemical reactions, Fe_2_O_3_, MoO_3,_ and iron sulphate facilitated lubrication [35,49,50]. The MoO_3_, a soft material formed after tribo-oxidation, helped in adherence of the MoS_2_ nanosheets on the surface [35,50]. The iron sulphate enhanced the capacity of adsorption of the nanosheets [50] on the sliding surfaces, thus decreasing the shearing force and boosting the lubrication.

## 4. Conclusions

The nanoparticles of zirconia (ZrO_2_) and 10%, 20%, and 30% cerium-doped zirconia (ZCO-1, ZCO-2 and ZCO-3, respectively) were prepared by the auto-combustion method. The rGO prepared by modified Hummer’s method was used to synthesize binary nanohybrids of zirconia (ZrO_2_@rGO) and 20% cerium-doped zirconia (ZCO-2@rGO). Since 10% and 30% cerium-doped zirconia yielded relatively poor results in tribology test, their nanocomposites were not prepared. Further, MoS_2_ nanosheets were prepared by hydrothermal method using ammonium heptamolybdate. These nanosheets were used to prepare ternary nanohybrids ZrO_2_@rGO@MoS_2_ and ZCO-2@rGO@MoS_2_ with the help of the microwave. The as-prepared nanosheets, nanoparticles, and nanocomposites were characterized by the state-of-the-art techniques such as Raman, FT-IR, SEM/HR-SEM with EDX, TEM/HR-TEM, and powder XRD. The constituents of the nanohybrids were bonded to each other by the noncovalent type of interactions. The dispersions of nanohybrids in the base lube were tested for stability using UV/visible spectroscopy and were found to be almost stable even beyond 48 h. The tribological activity of the synthesized additives in base oil was examined based on parameters, MWD, COF, load-carrying capacity, and wear rates gathered from ASTM-D4172 and ASTM-D5183 tests using four-ball tribo-tester at the optimized concentration, 0.125% w/v. The activity of different additives in paraffin oil was obtained to endorse the order mentioned below:

ZCO-2@rGO@MoS_2_ > ZrO_2_@rGO@MoS_2_ > ZCO-2@rGO > ZrO_2_@rGO > ZCO-2 > MoS_2_ > ZrO_2_ > rGO > PO

From the above studies, it is clear that the tested nanoparticles and nanosheets exhibit sufficiently good tribo-activity. Nevertheless, the activity inflates when binary nanohybrids are tested. In the case of ternary nanohybrids, the activity data surmount all other additives. Unquestionably, strong synergistic interaction between nanoparticles and nanosheets rendered such a high order of activity. The SEM and AFM studies of the worn surface validate the given order. Based on XPS studies, a plausible mechanism of lubrication includes the formation of tribofilm composed of adsorbed rGO, MoS_2,_ zirconia, ceria, and the products of tribochemical reactions, Fe_2_O_3_, MoO_3,_ and iron sulphate. Thus, the ternary nanohybrids in general and ZCO-2@rGO@MoS_2_, in particular, may be put forward as prospective antiwear and antifriction agents for lubrication systems.

## Figures and Tables

**Table 1:** Wear-rate for paraffin oil (PO) in the presence and absence of nano additives for 60 min test duration at 392 N applied load.

S/N	Lubricants	Wear Rate (10^−4^ × mm^3^/h)	
		Running-In	Steady-State
1	PO	84.482 ± 3.556	53.326 ± 3.359
2	rGO	31.074 ± 2.292	18.784 ± 3.236
3	ZrO_2_	24.664 ± 3.092	13.080 ± 2.125
4	MoS_2_	20.724 ± 4.227	11.080 ± 0.970
5	ZCO-2	17.524 ± 1.484	7.080 ± 0.969
6	ZrO_2_@rGO	14.864 ± 1.270	5.680 ± 0.162
7	ZCO-2@rGO	10.728 ± 2.321	2.140 ± 0.497
8	ZrO_2_@rGO@MoS_2_	6.640 ± 2.102	1.600 ± 0.439
9	ZCO-2@rGO@MoS_2_	3.543 ± 1.039	0.588 ± 0.263

## Supplementary Materials

The following are available online at https://www.mdpi.com/2079-4991/10/4/707/s1, Figure S1: EDX elemental mapping of (a) ZCO-2@rGO, and (b) ZCO-2@rGO@MoS_2_ nanomaterials, Figure S2: (a) Dispersion stabilities of base oil containing rGO, ZrO_2_, MoS_2_, ZCO-2, ZrO_2_@rGO, ZCO-2@rGO, ZrO_2_@rGO@MoS_2_ and ZCO-2@rGO@MoS_2_ studied by UV-vis spectrophotometry (inset showing a decrease in absorbance of 320 nm band against time). (b) Optical photographs of (I) plain PO, and PO with dispersed nano additives (II) rGO, (III) ZrO_2,_ (IV) MoS_2_, (V) ZCO-2, (VI) ZrO_2_@rGO, (VII) ZCO-2@rGO, (VIII) ZrO_2_@rGO@MoS_2_, and (IX) ZCO-2@rGO@MoS_2_ at zero time and after 48 hours, Figure S3: Determination of running-in wear rate by varying mean wear volume with time (h) for paraffin oil containing (0.125% w/v) nanoadditives at 392 N applied load, Figure S4: Determination of steady-state wear rate by varying mean wear volume with time (h) for paraffin oil containing (0.125% w/v) nanoadditives at 392 N applied load, Figure S5: Elemental mapping of worn surface lubricated with the blend of ZCO-2@rGO@MoS_2_ nanohybrid in paraffin oil at 392 N applied load.
