# Supplementary material for: Synergistic Tribo-Activity of Nanohybrids of Zirconia/Cerium-Doped Zirconia Nanoparticles with Nano Lamellar Reduced Graphene Oxide and Molybdenum Disulfide
Source: Nanomaterials (Basel). 2020 Apr 8;10(4):707. doi: 10.3390/nano10040707 (PMC7221905; doi:10.3390/nano10040707)
Supplement: Supplementary file 1 [file nanomaterials-10-00707-s001.pdf]

nanomaterials

Supporting Information

Synergistic Tribo-Activity of Nanohybrids of Zirconia/ Cerium-Doped Zirconia Nanoparticles with Nano Lamellar Reduced Graphene Oxide and Molybdenum Disulfide

Dinesh K. Verma[1,2], Nivedita Shukla[1], Bharat Kumar[1], Alok K. Singh[1], Kavita[1], Mithilesh Yadav[2], Kyong Yop Rhee[3]* and Rashmi B. Rastogi[1]*

[1] Department of Chemistry, Indian Institute of Technology, Banaras Hindu University, Varanasi 221005, India; dineshkv.rs.chy15@iitbhu.ac.in (D.K.V.); niveditashukla.rs.chy17@itbhu.ac.in (N.S.); bharatkr.rs.chy16@itbhu.ac.in (B.K.); alokkrsingh.rs.chy17@itbhu.ac.in (A.K.S.); kavita.rs.chy17@itbhu.ac.in (K.)

[2] Department of Chemistry, Prof. Rajendra Singh (Rajju Bhaiya) Institute of Physical Sciences for Study & Research, V.B.S. Purvanchal University, Jaunpur-222003, India; dryadavin@gmail.com (M.Y.)

[3] Department of Mechanical Engineering, College of Engineering, Kyung Hee University, Yongin 446-701, Korea

*Correspondence: rheeky@khu.ac.in (K.Y.R.); rashmi.apc@iitbhu.ac.in (R.B.R.);
Tel.: +82-312012565 (K.Y.R.); +91-9415817400 (R.B.R.)

S1. Experimental Details

For each experiment arithmetic mean of the diameter of each ball (d_1, d_2 and d_3) was taken as given by equation (**1**). The three stationary balls were not disturbed while taking the readings and the wear scar diameter was taken by Image acquisition system.

Tribological Parameters

Mean Wear Scar Diameter (MWD)

$$d = \frac{d_1 + d_2 + d_3}{3} \quad \textbf{(1)}$$

Mean Wear Volume (MWV)

Wear volume, $$V = \frac{\Pi\, d_0^4}{64\, r}\{(\frac{d}{d_0})^4 - (\frac{d}{d_0})\} \quad \textbf{(2)}$$

Hertzian diameter, $$d_0 = 2(\frac{3\,Pr}{4E})^{\frac{1}{3}} \quad \textbf{(3)}$$

Where, $$\frac{1}{r} = \frac{1}{r_1} + \frac{1}{r_2}$$

$$\frac{1}{E^*} = \frac{1 - v_1^2}{E_1} + \frac{1 - v_2^2}{E_2}$$

Where, E^* = Resultant modulus of elasticity

v = Poisson's ratio

r = Radius of steel ball

$E_1 = E_2 = 206$ GPa

$v_1 = v_2 = 0.3$

P = Actual load in Newton on each of the three horizontal balls that is 0.408 times of applied load.

Wear Rate

Overall, running-in and steady-state wear rates have been calculated on the basis of observed mean wear volume data at different time intervals. Mean wear volumes at different times (0.25, 0.50, 0.75, 1, 1.25 and 1.5 h) for each experiment were plotted with time and a linear regression model was fitted on the points including origin to find out overall wear rate.

$$\frac{V}{l} = K\frac{P}{H}$$

V = mean wear volume

l = sliding distance (2πr.N)

K = wear coefficient

H = hardness of steel ball (59-61 HRC)

P = applied load (0.408x392N)

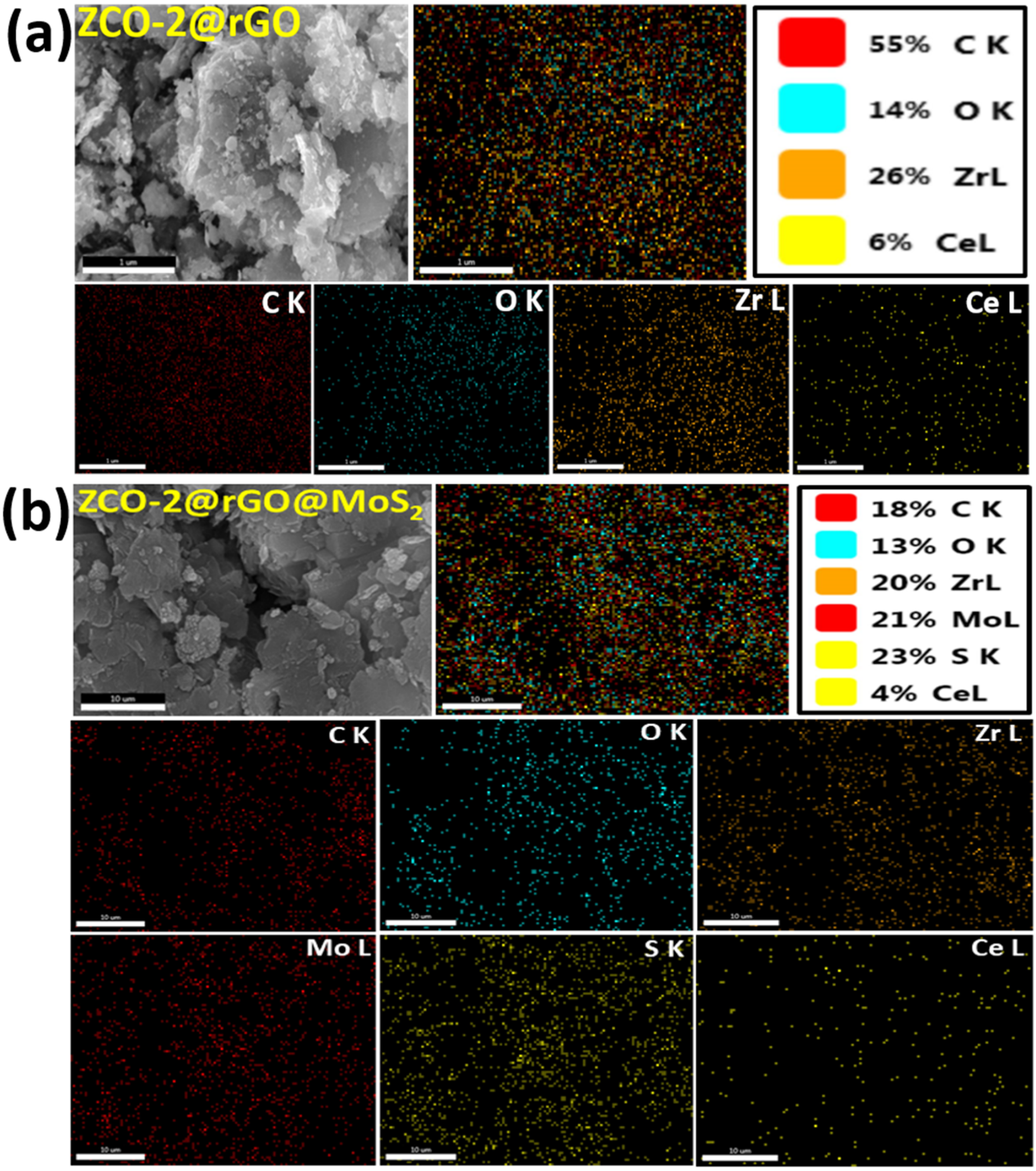

Figure S1. EDX elemental mapping of **(a)** ZCO-2@rGO, and **(b)** ZCO-2@rGO@MoS_2 nanomaterials.

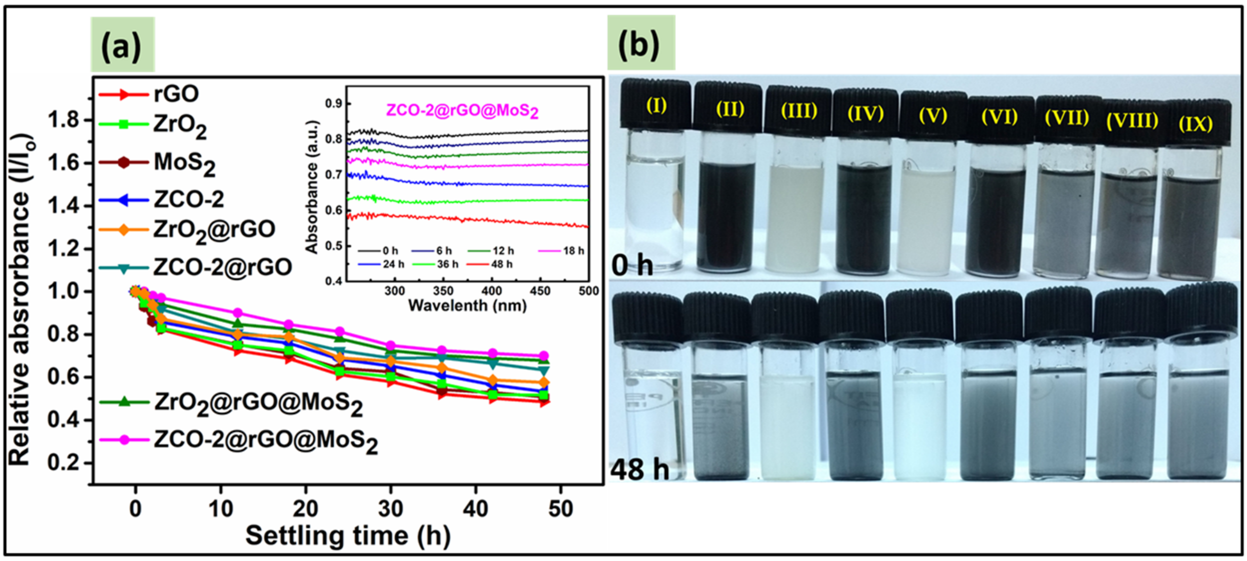

Figure S2. **(a)** Dispersion stabilities of base oil containing rGO, ZrO_2, MoS_2, ZCO-2, ZrO_2@rGO, ZCO-2@rGO, ZrO_2@rGO@MoS_2 and ZCO-2@rGO@MoS_2 studied by UV-vis spectrophotometry (inset showing a decrease in absorbance of 320 nm band against time). **(b)** Optical photographs of (I) plain PO, and PO with dispersed nano additives (II) rGO, (III) ZrO_2, (IV) MoS_2, (V) ZCO-2, (VI) ZrO_2@rGO, (VII) ZCO-2@rGO, (VIII) ZrO_2@rGO@MoS_2, and (IX) ZCO-2@rGO@MoS_2 at zero time and after 48 hours.

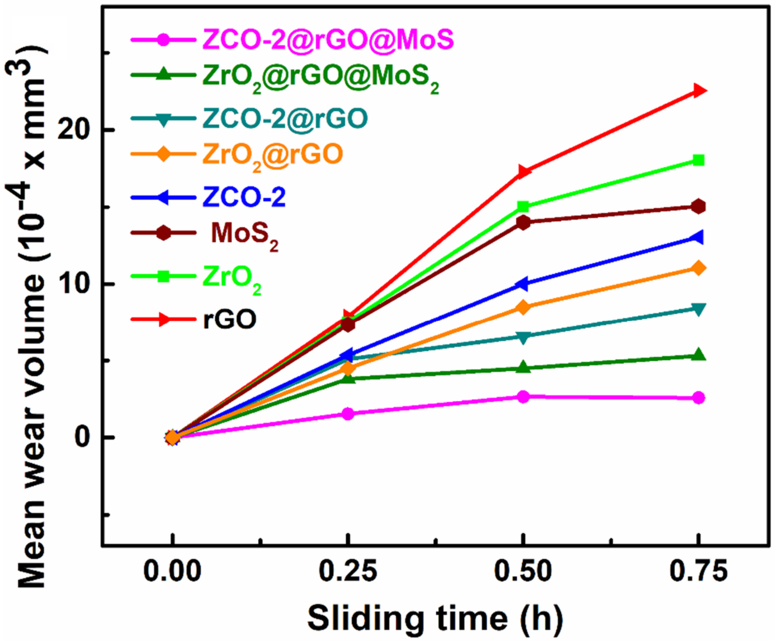

Figure S3. Determination of running-in wear rate by varying mean wear volume with time (h) for paraffin oil containing (0.125% w/v) nanoadditives at 392 N applied load.

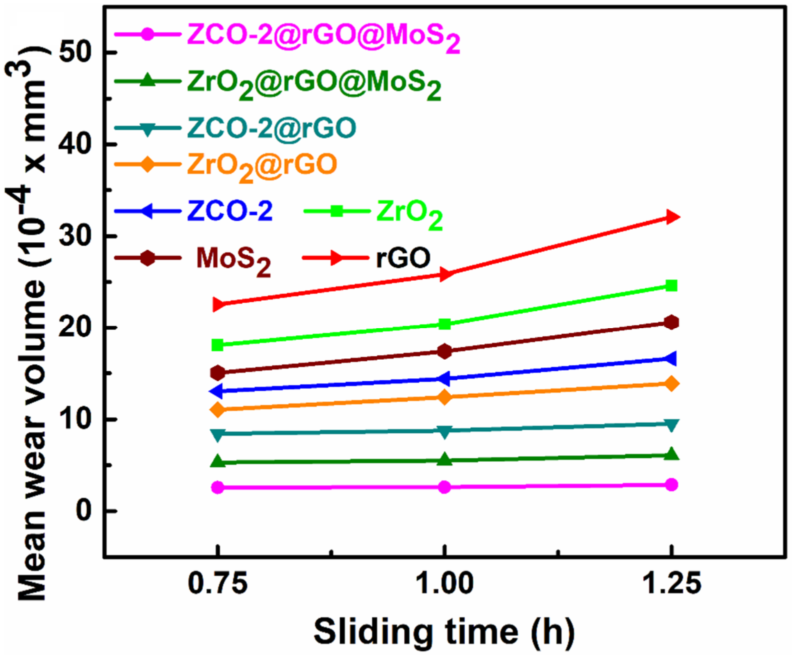

Figure S4. Determination of steady-state wear rate by varying mean wear volume with time (h) for paraffin oil containing (0.125% w/v) nanoadditives at 392 N applied load.

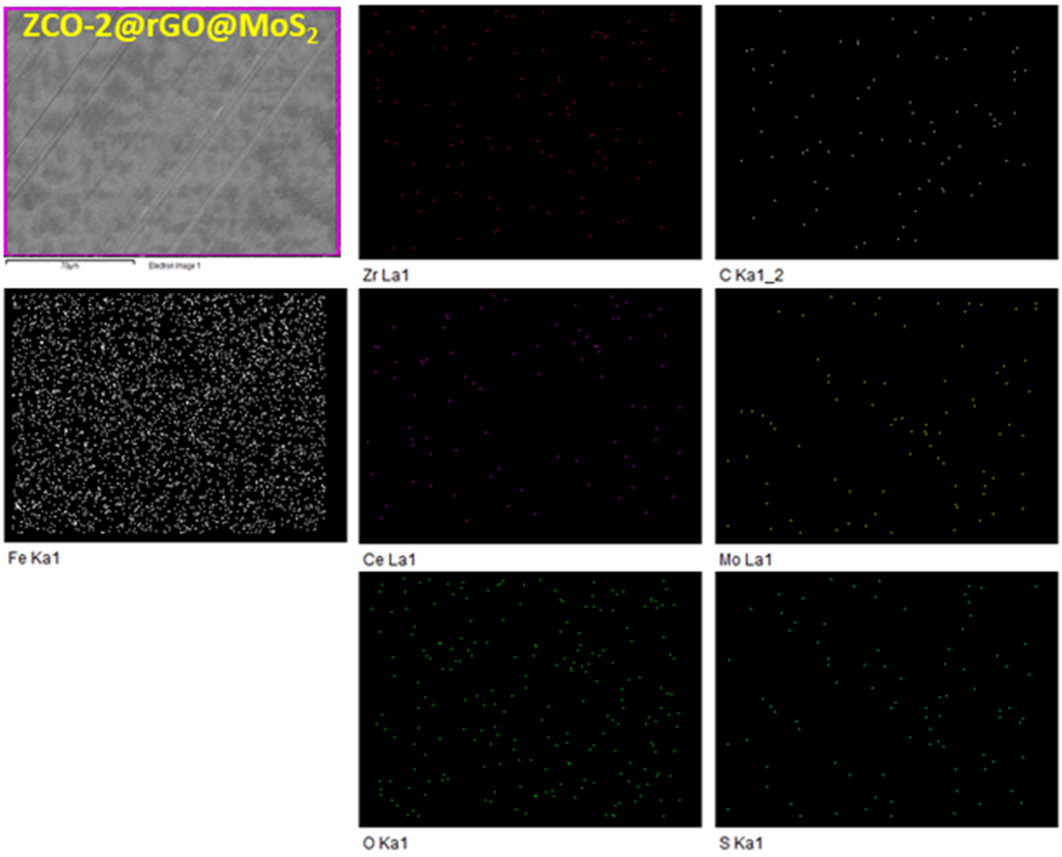

Figure S5. Elemental mapping of worn surface lubricated with blend of ZCO-2@rGO@MoS_2 nanohybrid in paraffin oil at 392 N applied load.
